# Reasons for bias in ambulance clinicians’ assessments of non-conveyed patients: a mixed-methods study

**DOI:** 10.1186/s12873-022-00630-8

**Published:** 2022-05-06

**Authors:** Helena Johansson, Kristin Lundgren, Magnus Andersson Hagiwara

**Affiliations:** 1 Falck Ambulans Östergötland, Faculty of Caring Science, Work Life and Social Welfare, University of Borås, SE-501 90 Borås, Sweden; 2 Ambulanssjukvården Region Jönköpings län, Faculty of Caring Science, Work Life and Social Welfare, University of Borås, SE-501 90 Borås, Sweden; 3 Centre for Prehospital Research, Faculty of Caring Science, Work Life and Social Welfare, University of Borås, SE-501 90 Borås, Sweden

**Keywords:** Ambulance nurse, Non-conveyance, Patient safety, See and treat

## Abstract

**Background:**

The number of ambulance assignments and the influx of patients to the emergency departments (EDs) in Sweden have increased in recent years. This is one reason the protocol for prehospital emergency care was developed around referring patients for non-conveyance, either through the see-and-convey elsewhere approach or through the see-and-treat approach. However, this protocol has led to challenges in patient assessments.

This study aimed to investigate the underlying causes of patient harm among those referred for the see-and-treat approach by the emergency medical services.

**Methods:**

This three-phase study involved a mixed-methods design. Cases of injuries, internal investigations and incident analyses of referrals for the see-and-treat approach in two regions in south eastern Sweden from 2015 to 2020 were examined using qualitative content analysis. This qualitative analysis was the basis for the quantitative analysis of the ambulance records. After the qualitative analysis was completed, a review protocol was developed; 34 variables were used to review 240 randomly selected ambulance records logged in 2020, wherein patients were referred for the see-and-treat approach. Finally, the review results were synthesised.

**Results:**

The qualitative analysis revealed three common themes: ‘assessment of patients’, ‘guidelines’ and ‘environment and organisation’. These results were confirmed by a medical journal review. Shortcomings were found in the anamnesis and in the number of targeted examinations performed. The checklist for referring patients for the see-and-treat approach and the information sheet to be provided to the patients were not used. In 34% of the ambulance records examined, the EMS clinicians deviated from the current guidelines for a see-and-treat referral.

**Conclusions:**

The results indicated that the low adherence to guidelines and the patient assessment deviating from the protocol put patients at risk of being harmed during a see-and-treat referral.

Measures are needed to guarantee a safe assessment of an increasing number of patients who are referred for the see-and-treat approach, especially the multi-sick elderly patients.

**Supplementary Information:**

The online version contains supplementary material available at 10.1186/s12873-022-00630-8.

## Background

Prehospital emergency care, both nationally and internationally, has developed rapidly, and the number of ambulance missions has gradually increased over time [1]. The protocol for prehospital emergency care has changed from assessing, treating and transporting patients to the emergency department (ED) to assessing, treating, and then referring patients to the appropriate level of care. This change has led to the emergence of the concept of non-conveyance, wherein some patients are not transported to the ED. The referral options for non-conveyance are as follows: *see and convey elsewhere*, wherein a patient is transported to a healthcare facility other than the ED, and *see and treat*, wherein a patient is not transported elsewhere after the on-site examination and treatment [2]. When a patient is suitable for non-conveyance, the important variables to take note of are age, gender, previous illnesses, geographical location, and signs and symptoms. The time of the day also seems to be an important variable, as many patients are found to be suitable for non-conveyance at night time [3, 4]. The majority of patients suitable for non-conveyance display unspecific symptoms based on the assessment made by the emergency medical service (EMS) clinicians, and most of them are women [3, 5]. The three other top conditions that render patients suitable for non-conveyance are abdominal pain, dyspnoea and chest pain [3, 5, 6]. Renewed healthcare contact within 72 h among non-conveyance patients has been reported to be approximately 10%; of these cases, 46% were hospitalized [6]. Patients who are transported to the ED are older than the non-conveyance patients [7]. In Sweden, non-conveyance is guided by guidelines and triage systems, both of which apparently differ among organisations. What is common among organisations, however, is that a triage system is used to determine whether a patient is suitable for non-conveyance.

Non-conveyance presents challenges to EMS clinicians [3, 7, 8], as it can increase the risk of patient harm. In particular, patients with time-sensitive conditions face the risk of receiving delayed causal medical treatment [3, 9, 10]. There are many reasons why patients with time-sensitive conditions are referred for the see-and-treat approach by the EMS. One reason is when a patient refuses to be transported to the ED [3], but the most common reason is probably the bias in prehospital assessment and decision making [11]. A study that compared EMS clinicians’ field assessment with the final assessment in hospital has found that EMS missed time-sensitive diagnoses in 12% of the cases [12]. This discrepancy can be attributed to several reasons. In some cases, a patient’s condition may worsen over time; moreover, EMS clinicians’ ability to perform advanced assessment are reduced due to the lack of resources that can only be found in hospitals, and medical support is limited in the prehospital emergency setting. Low adherence to guidelines and protocols is also a problem in prehospital emergency care [13]. The reason for low compliance is possibly the poorly adapted guidelines, but there are several other potential reasons [14].

Although guidelines and triage systems are in place, not all assessments of non-conveyance patients are performed in accordance with these guidelines, which can affect prehospital patient safety. Two studies [6, 7] have found a 14–23% deviation from the guidelines when handling non-conveyance cases. Such discrepancies are mainly attributed to the organisation’s triage system not being used or incorrectly used or to vital parameters not being noted or being applied incorrectly. It was against this background that the current study aimed to investigate the underlying causes of patient harm among those referred for the see-and-treat approach by the EMS.

## Methods

### Design

This three-phase study was performed using a mixed-methods design [15]. The first phase involved a qualitative content analysis, whereas the second phase involved a quantitative journal review. In the third phase, the obtained results were synthesised.

### Population and settings

This study was conducted in two regions in southeastern Sweden with 830,000 inhabitants. These regions have 101,000 ambulance assignments per year. A total of 45 ambulances and 3 transport ambulances are distributed in 22 stations. In both regions, the EMS organisations have been referring patients to different levels of care other than the ED since 2015. In the same year, guidelines and decision support for patient referrals were established. The guidelines for referral to the see-and-treat and to the see-and-convey elsewhere approaches are similar for the two regions.

Since 2005 in Sweden, ambulances have been manned by at least one registered nurse (RN). In Sweden, RNs undergo 3 years of training before being conferred with a bachelor’s degree. Currently, no requirements have been set for an RN to become a specialist nurse in prehospital emergency care (except for 1 year additional training). An ambulance team in Sweden may consist of two RNs with or without specialist training or an RN together with emergency medical technicians (EMTs) with 1 year prehospital training. The RN independently administers around 30 different drugs according to guidelines and general delegation [16].

The guidelines followed in the two regions when referring patients for the see-and-treat approach differ. In one region, a checklist and an information sheet must be provided to a patient. In the other region, the guidelines emphasize the importance of a consent from a patient prior to him/her being referred for the see-and-treat approach; moreover, a medical support (an emergency doctor who is on call at the ED) may be contacted for advice on patient assessment.

Triage decisions in the included organisations are premised on the triage system called Rapid Emergency Triage and Treatment System (RETTS) [17], which, in turn, is based on the Vital Parameters (VP) and Emergency Signs and Symptoms (ESS) codes. The codes consist of numbers that represent a symptom (e.g. chest pain), and each code is further expanded to indicate different degrees of severity. Also, there may be suggestions for targeted examinations (e.g. electrocardiogram (ECG)). Both VP and ESS are presented in different colours (red, orange, yellow and green), where red indicates the most serious condition. The colour representing the highest degree for VP or ESS applies as the triage colour. According to the guidelines followed in the participating organisations, only patients with a green triage colour may be referred for the see-and-treat approach.

For the second part of this study, namely, the journal review, EMS records were obtained from the region that had reported the higher number of referrals for the see-and-treat approach and had the majority of its reported cases classified as lex Maria cases (HSLF-FS 2017: 41).

According to the Swedish Patient Safety Act (SFS 2010: 659), a care provider is obliged to investigate and report incidents that could have caused a serious care injury to the Swedish Health and Care Inspectorate (IVO) in accordance with the lex Maria Act (HSLF-FS 2017: 41). The purpose is to clarify the course of events and the influencing factors, as well as to suggest measure that can prevent similar events from recurring. IVO is responsible for reviewing a care provider’s investigation and for determining whether the investigation adheres to the applicable laws, in which instance it will close the case. An event analysis can be part of an internal investigation of a lex Maria report. Event analysis and investigation on deviations form part of the systematic improvement work, which is a prerequisite for high patient safety. Sweden’s Municipalities and Regions (SKR) has produced a handbook for risk analysis and incident analysis in health and medical care [18]. The analysis methods aim to identify shortcomings in organisations that may put patient safety at risk.

### Data collection and selection

Given that the guidelines for referring patients for the see-and-treat approach was established in 2015, the lex Maria reports concerning prehospital emergency care in the included regions for the years 2015–2020 were requested from IVO. Five of the ten lex Maria reports were related to the EMS clinicians’ patient assessment when referring patients for the see-and-treat approach, and they were included in this study. Five internal investigations and one incident analysis were received from IVO. Another three incident analyses were obtained from the EMS organisation of one concerned region. Moreover, EMS medical records related to the lex Maria cases were obtained. The Lex Maria cases were subsequently examined.

EMS medical records were obtained by conducting searches in Paratus, the records system of the included regions. The inclusion criterion was an ambulance record from 2020 with the assignment type ‘primary assignment care level—see and treat/treatment on-site’. Patients aged below 18 years were excluded, as they are not covered by the current guidelines for referring patients for the see-and-treat approach.

**Fig. 1:**
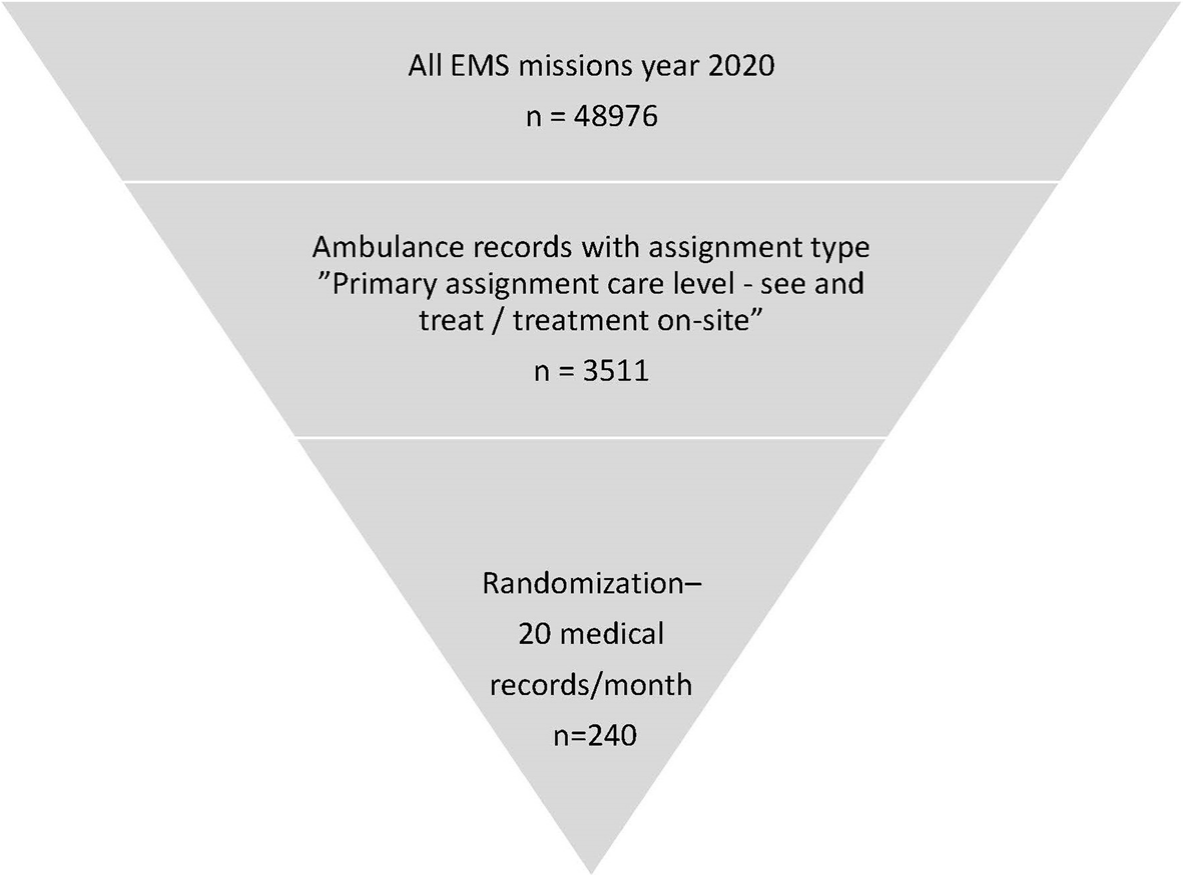
Flowchart depicting the selection of medical records

The search results comprised 3511 EMS medical records. The number of EMS medical records varied from 185 to 365 per month. In addition, 240 EMS medical records were randomly selected using a random number generator—20 medical records per month—to get an even distribution over the year (Fig. 1).

### Analysis

#### Phase 1: Lex Maria cases

In accordance with the observations made by Graneheim and Lundman (2004) [19], a qualitative content analysis was applied to the lex Maria cases with associated EMS medical records, internal investigations and event analyses to obtain a deeper understanding of the cases.

The texts were read carefully several times to obtain an overall picture of the cases. Sentences related to the study aim were extracted and pooled, forming the basis for further analysis. The text was divided into meaning units, which were then condensed and abstracted into codes. The codes were first sorted into subthemes before being combined into three themes [19] (Table 1).

**Table 1:** Examples of the analysis process

Unit of meaning	Condensed unit of meaning	Code	Subtheme	Theme
A deviation from normal values was the pulse rate of 110 beats/minute; the patient was thus recommended for the see-and-treat approach	The RETTS colour was yellow, but the patient was recommended for the see-and-treat approach	Incorrect referral by the EMS clinician	RETTS colour	Guidelines
Deficiencies in anamnesis and in the examination of patients, as well as lack of differential diagnoses, leading to premature closure	Unstructured interview technique and patient assessment	Lack of compliance to the advanced medical life-support system	Prehospital assessment of adult patients	Patient assessment
Lack of teamwork and shared decision making, which were a possible contributing factor to the shortcomings in patient assessment	Lack of teamwork and shared decision making impacts patient assessment	Negative workplace culture	Workplace culture	Environment and organisation

#### Phase 2: EMS medical records

After the completion of the content analysis, which showed that in the lex Maria cases there were shortcomings in the anamnesis and focused examinations and that the guidelines for the see-and-treat referrals were not followed, a medical record review protocol was developed using 34 variables. The variables represented anamnestic data, such as symptoms, allergies, current medications, previous illnesses, elimination, meals, what preceded the symptom onset, times, vital parameters, RETTS colour and prehospital assessed condition, as well as the variables in the see and treat guidelines.

The review began with a joint assessment of 10 EMS medical records. The remaining records were divided and reviewed individually by two authors (HJ and KL). Data were entered into an Excel file and then transferred to the IBM SPSS Statistics Data Editor. For the demographic data, the number and percentage, as well as the mean and standard deviation, were used.

#### Phase 3: synthesis

The results of the initial analyses were synthesised to determine which of the observed risks in the lex Maria cases recurred in the journal review.

The synthesis used the triangulation employed by Yin [20] to find similarities and contradictions between data sources. This was performed by HJ and KL by asking questions such as ‘Can we see the deviations from the guidelines in the medical records described in the Lex Maria cases?’ and ‘Are there similarities and/or differences in the assessment process in both the lex Maria cases and in the medical records?’. After the primary triangulation, all authors discussed the results until a consensus on the synthesis results was reached.

## Results

### Lex Maria cases

The five Lex Maria cases consisted of patients suffering from myocardial infarction, severe pancreatitis, sepsis, ruptured aortic aneurysm and acute pancreatitis that eventually led to myocardial infarction. The patients included three women and two men aged 69–79 years, with an average age of 73.4 years.

In the content analysis, three overarching themes emerged: *patient assessment, guidelines* and *environment and organisation.*

### Patient assessment

This theme describes different aspects of EMS clinicians’ patient assessment. It illustrates the use of digital decision support, patients’ consent to be referred for the see-and-treat approach, and relatives’ confidence in EMS assessment.

#### Prehospital assessment of adult patients

The analysis consistently showed that the current guidelines for referral were not followed.

The primary assessment was documented in most cases through fixed pre-selection in the journals, but it was not commented on in any of the journal texts. Thus, it is difficult to assess the accuracy of the primary assessment performed for each case.

In the two internal investigations, shortcomings were found in the anamnesis and examination. The anamnesis did not follow any structure; some parts were missed or not documented. For example, the main symptoms were not described in detail, and previous diseases were sparsely documented. In all event analyses, the lack of differential diagnoses contributed to the patients being referred for the see-and-treat approach, as the primary diagnosis was less serious, such as gastroenteritis.

Shortcomings in the focused examinations were also noted based on the patients’ symptoms. Pulmonary auscultation, ECG, pain assessment, plasma glucose determination and neurological and abdominal examinations were some of the focused examinations that were not performed in many cases, despite being included in the current guidelines.

#### Transient symptoms

In three cases, the patients’ symptoms subsided or disappeared completely when the ambulance arrived. These symptoms included transient chest pain, abdominal pain, nausea, dizziness, cold sweats and vomiting. The EMS medical record analysis and the event analyses demonstrated that more serious differential diagnoses were not considered and thus were not investigated further.

#### Digital decision support

As an aid used by EMS clinicians’ during patient assessment, a portable digital decision support is handed to a patient; this device contains the triage guidelines, treatment guidelines, checklists and decision support for the referral for the see-and-treat approach. The referral guidelines state that the emergency bag and the digital decision support must always be brought to the patient. The review results showed that such a practice was normally neglected, indicating that the EMS clinicians had overlooked important information.

#### Consent to be referred for the see-and-treat approach

Two out of five patients gave a consent to be referred for the see-and-treat approach, to which the patients and relatives were reportedly satisfied. In one specific case, the patient was anxious about entering the ED when the ambulance arrived at the patients’ place, but the patient was happy to remain at home after being examined and given medications. In several cases, EMS clinicians ensured that relatives were present at home to provide patient care. Relatives were also advised by the EMS clinicians to call the dispatch centre if their patient’s condition worsened.

#### Confidence in the EMS assessment

The internal investigations revealed that the patients’ relatives largely trust the EMS clinicians’ assessment. If the condition was assessed as less serious, relatives depended on the EMS clinicians’ assessment The assessments of the lex Maria cases showed that the EMS clinicians advised the patients to wait at home, to perform self-care and to seek care again if there is no improvement or when their condition worsens. However, two event analyses revealed that the EMS clinicians’ decision led to delayed care.

### Guidelines

This section describes the EMS clinicians’ compliance to guidelines, as well as their use of the checklist and information sheet when referring patients for the see-and-treat approach. Also, the use of medical support in the concerned lex Maria cases is presented.

#### RETTS colour

Patients who triaged yellow, orange or red according to the RETTS were not referred for the see-and-treat approach as an alternative management approach. The patients who triaged yellow in four Lex Maria cases were advised to be cared for at home. In the fifth case, information about RETTS colour was missing. The review also showed that patients received very low RETTS scores; this is because previous risk factors, such as myocardial infarction, stroke, or hypertension, were not taken into account in the prehospital assessment.

#### Checklist for referral for the see-and-treat approach

The checklist for a see and treat referral was not used in any of the lex Maria cases. Had the EMS clinicians used the checklist, it would have been clear that none of the patients was suitable for the see-and-treat approach. Filling out the checklist was mandatory when advising patients to be treated at home.

#### Form/information sheet for the referred patients

The form/information sheet for the referred patient was not provided to the patients in the lex Maria cases. The event analyses showed that the ambulance nurses often gave information verbally instead.

#### Medical support

The internal investigations have found that physicians must be contacted should EMS clinicians deviate from the guidelines or should a patient does not wish to be brought to the hospital. Deviation from the guidelines could be justified when a personnel with higher medical competence was contacted. In the concerned lex Maria cases, however, no consultations were made.

### Environment and organisation

This theme describes how the workplace culture, interaction with the ED, and information/previous experiences affect prehospital patient assessment. It also presents the risk prevention measures taken after the review of the lex Maria cases.

#### Workplace culture

In several event analyses, workplace culture was found to be a possible contributing factor to the shortcomings in assessments. In all cases, it was difficult to determine whether this problem exists, and its challenging to assess its possible impact. The internal investigations have shown that guidelines in the organisation have been assessed to be appropriate, and the reasons for deviations have not been clarified.

It was important for the ambulance crew to work as a team and make a joint assessment of a patient’s condition. Despite this, the incident analyses have found that only one ambulance crew member was involved in the interview and examination of patients. It was also observed that the EMS clinicians did not consider that the digital decision support was necessary to help their patients.

#### Interaction with the ED

The EMS clinicians have expressed concerns that the ED staff would get annoyed at the ‘unnecessary’ transport of a patient. They assumed that if they handover a patient with, for example, abdominal pain but without any other symptoms, they would end up having a heated discussion with the ED personnel. This concern was based on several occasions when an ED personnel questioned the EMS clinicians, insinuating the latter’s lack of knowledge on prehospital assessment and on the EMS care protocol. The importance of a permissive culture between organisations was emphasized in several internal investigations.

The results also showed the EMS clinicians’ lack of knowledge on the protocols in the ED and on the measures to be performed on patients in specific conditions. For example, when handling a patient with abdominal pain and who had been vomiting, they mentioned to the patients’ relatives that their patient will only be observed when brought to the hospital and will not be further assessed.

#### Information and previous experience

Information from the dispatch centre was a risk factor for an accurate patient assessment. When interpreting a dispatch text, information could be overlooked, and whether the patient would provide the same information when the ambulance arrives is uncertain. Moreover, a dispatch text may lead EMS clinicians to come up with a preconceived opinion about a patient’s condition and the level of care offered.

According to the event analyses, the EMS clinicians have had previous experience handling patients with similar cases. For example, the EMS clinicians were fixated with the idea that the symptoms were related to gastroenteritis and placed too much weight on the previous experiences of patients with a similar case, affecting the EMS assessment.

Lack of access to a patient’s medical record sometimes lead EMS clinicians to obtain additional information from, for example, the ED personnel. This can adversely impact the quality of prehospital assessments. Information such as the patient previously applying for a similar condition and being later assessed as benign implied that the EMS clinicians assumed that the relevant symptoms remained unchanged.

#### Risk prevention measures

The internal investigations and the event analyses revealed several improvement measures that must be observed to avoid cases similar to the current lex Maria cases. All EMS clinicians were informed about these cases. The concerned EMS clinicians were informed of the incident and were invited for a corrective dialogue about patient assessment and treatment. In addition, they were required to review the guidelines.

EMS personnel were trained to ensure that patients are well informed. Advanced medical life support (AMLS) training became mandatory for all EMS personnel to ensure a structured anamnesis and examination technique. In this context, a digital decision support and documentation systems were installed in all ambulances in the investigated regions.

Educational initiatives, such as those for unstable coronary heart disease, abdominal pain, and infectious diseases with a focus on sepsis, have been implemented. The investigated regions introduced sepsis alarms, where EMS is a part of the care chain. Practical exercises in the form of abdominal examinations were carried out, along with a lecture on diagnostic limitations for prehospital-assessed conditions with symptoms involving the gastrointestinal tract.

Information has also been provided on workplace cultures and their impact on patient assessment and referral for the see-and-treat approach, which could justify the importance of EMS clinicians working as a team.

Training and improvement measures in the EMS records have been completed, and future improvements are being planned. For example, the development of an IT support has been suggested so that certain elements become mandatory. If that happens, it will not be possible to sign a journal without having made certain assessments or without having implemented certain measures.

### EMS medical record review

#### Demographics

More women than men were referred for the see-and-treat approach. The mean age of the referred patients was 59.3 years (SD 22.2) (Table 2).

**Table 2:** Demographic data over patients triaged to self-care by EMS

Variable	***N*** = 240
Female N (%)	131(54.6)
Men N (%)	109 (45.4)
Age years mean (SD)	59.28 (22.21)
**Priority from dispatch N (%)**	
Priority 1	88 (36.6)
Priority 2	142 (59.2)
Priority 3	10 (4.2)
**Documented vital signs mean (SD)**	
Respiration rate/min	17.03 (0.32)
Oxygen saturation/%	97.72 (0.23)
Pulse rate/min	82.47 (1.75)
Systolic blood pressure/mmHg	135.32 (2.44)
Diastolic blood pressure/mmHg	77.10 (1.37)
Body temperature/ °C	36.37 (0.64)
Plasma glucose/mmol/l	6.84 (0.33)
**Triage color vital signs N (%)**	
Green	217 (90.4)
Yellow	14 (5.8)
Orange	3 (1.3)
Red	2 (0.8)
No triage	4 (1.7)
**Triage color ESS N (%)**	
Green	154 (64.2)
Yellow	75 (31.3)
Orange	7 (2.9)
Red	3 (1.3)
Ni triage	1 (0.4)
**Triage color overall (RETTS)**	
Green	146 (60.8)
Yellow	77 (32.1)
Orange	10 (4.2)
Red	4 (1.7)
No triage	3 (1.2)

Of the patients referred for the see-and-treat at home approach, 37% from the dispatch centre were classified as priority 1(life threatening condition), 59% as priority 2 (potential serious condition), and 4% as priority 3 (non-serious condition). The two most common major complaints from the dispatch centre were chest pain and dyspnoea.

The ambulance nurse must identify the major complaints and the ESS scores that best correspond to the patient’s symptoms and signs. In the results, ESS 53 and ESS 4 were the most common symptoms. ESS 53 (non-specific disease, feeling sick/tired, health examination) was chosen in 10.8% of the cases, and ESS 4 (breathing problems/dyspnoea, breast pain when breathing) was chosen in 10% of the cases. Otherwise, different ESS values overlapped. For the see-and-treat approach to be recommended in accordance with the guidelines, all vital parameters must fall within the reference range for the RETTS green.

#### Prehospital assessment and interventions

A complete primary assessment of airway, breathing, circulation, disability and exposure (ABCDE) was performed and documented in 87% of the ambulance records. All vital parameters needed to be checked and documented at each patient assessment. SpO_2_ and disability were found to be the most controlled vital parameters, and diastolic blood pressure was the least controlled one. Pain assessment must also be performed before patients can be referred for the see-and-treat approach. However, the present results showed that pain assessment was documented in only 29.6% of the medical records. ECG was taken in approximately 33% of the cases, whereas EMS clinicians gave drugs in 12.9% of the assignments. The medications given were paracetamol, glucose and inhalations. A total of 21 variables were examined, of which 18 are mandatory according to the guidelines used when referring patients for the see-and-treat approach (Table 3). The remaining three may be mandatory, depending on the field diagnosis.

**Table 3:** Prehospital assessment and interventions among patients triaged to self-care

Assessment and interventions	Performed N (%)
**The first survey according to ABCDE**^a^	209 (87.1)
**Vital signs**	
Respiration rate^a^	229 (95.4)
Oxygen saturation^a^	232 (96.7)
Pulse rate^a^	231 (96.3)
Systolic blood pressure^a^	226 (94.2)
Diastolic blood pressure^a^	222 (92.5)
Disability^a^	232 (96.7)
Body temperature^a^	230 (95.8)
**The secondary survey, clinical history, and focused assessment**	
Major complaints^a^	240 (100)
Allergies^a^	115 (47.9)
Medication^a^	143 (59.6)
Previous history^a^	215 (89.6)
Nutrition, elimination^a^	71 (29.6)
What preceded the onset of symptoms^a^	236 (98.3)
Plasma glucose	75 (31.3)
ECG	80 (33.3)
Lung auscultation^a^	72 (30)
Abdominal examination	16 (6.7)
Neurological examination^a^	54 (22.5)
Pain assessment^a^	71 (29.6)
**Interventions**	
Drug administration	31 (12.9)
Physician contacted	51 (21.3)
Checklist completed^a^	16 (6.7)
Information sheet given to patient^a^	9 (3.8)
Self-care appropriate according to guideline	159 (66.3)

^a^ = Mandatory variable when referring to self-care

*ABCDE* Airway, Breathing, Circulation, Disability, Exposure

#### Compliance with the guidelines for the referral for the see-and-treat at home approach

Overall, the EMS clinicians did not adhere to the current guidelines on referring patients for the see-and-treat approach in 34% of the EMS medical records. The average age of those incorrectly referred for this approach was 64 years.

The results showed the lack of compliance among the EMS clinicians to the decision support and guidelines when recommending the see-and-treat at home approach.

Notably, 87.7% of the incorrectly referred patients had yellow ESS colour; 79% had green vital parameters, 15% had one or more yellow vital parameters, and 12.3% had a combination of at least yellow ESS colour and at least yellow VP colour. The most common vital parameters that deviated from the normal values were saturation, pulse and temperature.

The checklist for referral was used in only 6.7% of the records. Only 3.8% of the patients received an information sheet (Tables 3 and 4).

**Table 4:** Prehospital assessment and interventions in those patients where self-care was incorrect according to the guideline

Assessment and interventions	Performed ***N*** = 81 N (%)
**The first survey according to ABCDE**^a^	71 (87.7)
**Triage color overall (RETTS)**^a^	
Green	2 (2.5)
Yellow	73 (90.1)
Orange	4 (4.9)
Red	1 (1.2)
No triage	1 (1.2)
**Vital signs**	
Respiration rate^a^	76 (93.8)
Oxygen saturation^a^	79 (97.5)
Pulse rate^a^	78 (96.3)
Systolic blood pressure^a^	76 (93.8)
Diastolic blood pressure^a^	75 (92.6)
Disability^a^	79 (97.5)
Body temperature^a^	78 (96.3)
**The secondary survey, clinical history**	
Major complaints^a^	81 (100)
Allergies^a^	36 (44.4)
Medication^a^	55 (67.9)
Previous history^a^	75 (92.6)
Nutrition, elimination^a^	26 (32.1)
What preceded the onset of symptoms^a^	80 (98.8)
**Focused assessment**	
Plasma glucose	22 (27.2)
ECG	34 (42)
Lung auscultation^a^	29 (35.8)
Abdominal examination	5 (6.2)
Neurological examination^a^	16 (19.8)
Pain assessment^a^	25 (30.9)
**Interventions**	
Drug administration	15 (18.5)
Physician contacted	23 (28.4)

^a^ = Mandatory variable when referring to self-care

*ABCDE* Airway, Breathing, Circulation, Disability, Exposure

### Synthesis

The views regarding assessment that emerged in the event analyses are reflected in the review of the EMS medical records. Table 3 shows certain shortcomings in logging; moreover, it indicates that targeted surveys were carried out on a few occasions. As ESS 53 (non-specific disease, feeling sick/tired, health examination) is chosen most frequently, the EMS clinicians’ assessment does not seem to have been undertaken in any field diagnosis.

EMS clinicians deviated from the current guidelines on referring patients for the see-and-treat approach in 34% of the EMS medicalrecords reviewed. In all lex Maria cases, the patients were over 65 years old and were incorrectly referred for the above approach based on the guidelines. The EMS medcial recordreview showed that most incorrect referrals were made in patients aged 64 years and older.

Medical support was cited as an important resource and support in internal investigations. However, the medical record review shows that support was seldom used. Currently, there are no guidelines on medical support.

Lex Maria reports, internal investigations and event analyses should assume great importance for the development of prehospital care. The review of EMS medical records showed that despite the efforts exerted on training after the current cases were reported, the checklist for referring patients for the see-and-treat approach and the information sheet to be provided to patients were not used.

It is difficult to draw any conclusions as to whether EMS clinicians used the digital decision support and whether they sought the patients’ consent to be referred for the see-and-treat approach, as these pieces of information cannot be recorded accurately in the current medical records system.

## Discussion

The current findings showed that there are risks to patient safety, as EMS clinicians do not always conduct systematic clinical reasoning because they tend to deviate from the current guidelines and decision support. The low guidelines compliance put patients with time-critical conditions at risk as a result of the delayed causal care initiation. This is probably one of the most common threats to prehospital patient safety [10, 11, 21], and it becomes an even greater risk when associated with referrals for the see-and-treat approach [9].

Prehospital assessments are complicated, but they can be carried out well with experience, education, and awareness of clinical reasoning and risk of bias [22]. Nevertheless, an unacceptably high frequency of prehospital misdiagnosis remains to be a challenge [12]. Missed or delayed diagnoses are caused by both systematic and individual factors [23, 24].

During the qualitative content analysis, confirmation, expectation and representative bias may have existed when patients were referred for the see-and-treat approach instead of being transported to the ED. Based on previous experience and based on second-hand information, EMS clinicians systematically seek support to confirm their preconceived opinion about patients and their medical condition. This was also confirmed by a previous research [22].

A type of expectation bias was seen in the results when EMS clinicians based their decision concerning referring patients for the see-and-treat approach on inadequate knowledge of the ED protocols and on the previous treatments given by the ED personnel.

To avoid making cognitive mistakes that jeopardize patient safety, regular trainings and discussions on clinical decision making and biases should be a practice in EMS organisations [25, 26]. The dual-process theory is a model that renders clinical decision making apparent [27], wherein system 1 thinking is involved in arriving at quick and unconscious decisions, whereas system 2 thinking leads to a somewhat slower and more analytical decision making. This theory can be used to avoid the misjudgements that emerged in the result, which can be traced to the EMS clinicians choosing to take mental shortcuts in a system 1 mode of reasoning.

The review of the EMS medical records revealed that the EMS clinicians deviated from the current guidelines in one-thirds of the patients referred for the see-and-treat approach; this patient population is greater than previously observed [7]. Therefore, it is remarkable that considerably few lex Maria cases were reported in previous years. A suspicion that arises is that the threshold set to identify incorrect references in operations is too high, as only the most serious cases are being investigated. The fact that EMS clinicians deviate from the current guidelines may imply that patients do not receive adequate care. For this reason, it would be desirable to prepare guidelines and decision support that are based on the current research and are more compatible with reality [11, 13]. Compliance to prehospital recommendations regarding monitoring of vital parameters have been found to be higher than the compliance to treatment guidelines [13]. Research has shown that the EMS clinicians depend upon prehospital guidelines, but these guidelines are often poorly adapted to a certain context and are cumbersome to use [14]. Research has also shown that compliance to guidelines presented in a digital format with decision support that follow the prehospital assessment process is better compared with the compliance to paper-based guidelines [28, 29].

Checklists should support the clinical reasoning that minimizes the risk of cognitive bias decisions. They are particularly useful in uncertain conditions and when time is limited, as they contribute to a more analytical and systematic process [26]. If the checklist had been used in the prehospital assessments in cases that later became lex Maria reports, none of these patients should have been referred for the see-and-treat at home approach.

Not all assessed patients should be transported by ambulance; several of them should be recommended for another mode of transport to the ED or to primary care to avoid more serious differential diagnoses. Research [6] has shown that patients are not referred to heavily burdened primary care, which explains the high proportion of patients recommended for the see-and-treat approach in this study. It has been established that functional guidelines are required to make it possible for EMS clinicians to assess and refer patients to the most appropriate level of care [30].

The review of the EMS medical records showed that most patients who were recommended for the see-and-treat approach outside the guidelines were over 63 years old. This is supported by previous research showing that the elderly are triaged too low and do not receive the care they would have received based on the guidelines [6, 13]. When referring patients for the see-and-treat approach, the EMS clinicians in the lex Maria cases seem to have failed to reflect on whether the patients’ physical and mental health and life situation allow for it. As a suggestion, it should be emphasized in the referral guidelines that patients’ age and multi-morbidity are obviously a risk factor.

### Limitations

The number of lex Maria cases analysed is low, and this affects the conclusions that could be drawn from the results. However, these were the only available cases during the study period. The analysis of the EMS medical record review confirmed the results to some extent and strengthened the value of the results. The results from the lex Maria cases are based on the investigations made by IVO and by the EMS organisations, and it is difficult to assess the quality of these investigations. The investigations followed a process described by the SKR; however, whether the process was followed correctly is difficult to guarantee. It is important to understand that the results of the investigations could be subjective. With this background, transferability to other ambulance organisations that follow similar guidelines should be approached with caution.

This study chose a mixed-methods design based on two data sources. Supplementing this study with interviews among EMS clinicians on why compliance to the guidelines is quite low could provide a deeper understanding of the problem.

The review of the EMS medical records was based only on the documented information. Possibly, there were assessments and actions taken by the EMS clinicians that were not documented. This could have affected the results.

The two authors who conducted the qualitative analysis of the lex Maria cases are active EMS clinicians, and their analysis may have been influenced by their pre-understanding of things. This potential bias was discussed throughout the analysis to reduce the risk of impact.

## Conclusion

The present results indicated that the low adherence to guidelines and the patient assessment that deviates from a described structure may lead to patient harm in relation to the referral of patients for the see-and-treat approach. EMS organisations must increase training in patient assessment processes and improve the guidelines on patient assessment in general and on assessments when referring patients for the see-and-treat approach in particular. The low number of the lex Maria cases during the study period indicated that lex Maria is a poor indicator of quality.

## Supplementary Information

**Additional file 1.** 

## Data Availability

The datasets generated and/or analysed during the current study are not publicly available due to General Data Protection Regulation, GDPR but are available from the corresponding author on reasonable request.

## Abbreviations

ED: Emergency Department
EMS: Emergency Medical Service
RETTS: Rapid Emergency Triage and Treatment System
VP: Vital Parameters
ESS: Emergency Signs and Symptoms
IVO: Swedish Health and Care Inspectorate
SKR: Sweden’s Municipalities and Regions
ECG: Electrocardiogram
AMLS: Advanced Medical Life Support
ABCDE: Airway, Breathing, Circulation, Disability and Exposure
