# Supplementary material for: Reasons for bias in ambulance clinicians’ assessments of non-conveyed patients: a mixed-methods study
Source: BMC Emerg Med. 2022 May 6;22:79. doi: 10.1186/s12873-022-00630-8 (PMC9074185; doi:10.1186/s12873-022-00630-8)
Supplement: Supplementary file 1 — Additional file 1. [file 12873_2022_630_MOESM1_ESM.docx]

Supplemental file 1.

Review protocol

“Missing value”=333

- Age _________
- Gender _____       male=0, female=1
- Dispatch priority ________
- Case index from dispatch (SOS-alarm) _______
- ESS number________________

- ESS-color - green/yellow/orange/red green=0, yellow=1, orange=2, red=3
- VP-color - green/yellow/orange/red green=0, yellow=1, orange=2, red=3
- RETTS-color - green/yellow/orange/red green=0, yellow=1, orange=2, red=3
- Complete primary survey - yes/no no=0, yes=1
- Respiratory frequence - yes/no no=0, yes=1
- Spo2 - value
- Pulse - value
- Blood pressure - systolic
- Blood pressure – diastolic
- Reaction Level Scale (RLS) - value
- Temperature - value
- ECG - yes/no no=0, yes=1
- Blood glucose level - value
- Pain (VAS) - value
- Pulmonary auscultation - yes/no no=0, yes=1
- Abdominal examination - yes/no no=0, yes=1
- Neurological examination (FAST) - yes/no no=0, yes=1
- SAMPLE
  - S  - yes/no no=0, yes=1
  - A- yes/no no=0, yes=1
  - M- yes/no no=0, yes=1
  - P- yes/no no=0, yes=1
  - L- yes/no no=0, yes=1
  - E- yes/no no=0, yes=1

- Medicine given by EMS clinicians - yes/no no=0, yes =1
- Telephone support by doctor - yes/no no=0, yes=1
- Checklist for referral to see and treat completed - yes/no no=0, yes=1
- Form/Information sheet for the referred patient completed and submitted - yes/no no=0, yes=1
- Compliance to see and treat-guidelines (RETTS-colour green)- yes/no no=0, yes=1
